# Supragranular Pyramidal Cells Exhibit Early Metabolic Alterations in the 3xTg-AD Mouse Model of Alzheimer’s Disease

**DOI:** 10.3389/fncel.2018.00216

**Published:** 2018-07-18

**Authors:** Juliette Piquet, Xavier Toussay, Régine Hepp, Rodrigo Lerchundi, Juliette Le Douce, Émilie Faivre, Elvire Guiot, Gilles Bonvento, Bruno Cauli

**Affiliations:** ^1^UPMC Univ Paris 06, INSERM, CNRS, Neuroscience Paris Seine – Institut de Biologie Paris Seine (NPS – IBPS), Sorbonne Université, Paris, France; ^2^CNRS UMR 9199, Commissariat à l’Énergie Atomique et aux Énergies Alternatives (CEA), Département de la Recherche Fondamentale (DRF), Institut de Biologie François Jacob, Molecular Imaging Center (MIRCen), Université Paris-Sud, Université Paris-Saclay, Paris, France

**Keywords:** glucose uptake, glycolysis, pentose phosphate pathway, FRET imaging, single-cell RT-PCR

## Abstract

The impairment of cerebral glucose utilization is an early and predictive biomarker of Alzheimer’s disease (AD) that is likely to contribute to memory and cognition disorders during the progression of the pathology. Yet, the cellular and molecular mechanisms underlying these metabolic alterations remain poorly understood. Here we studied the glucose metabolism of supragranular pyramidal cells at an early presymptomatic developmental stage in non-transgenic (non-Tg) and 3xTg-AD mice, a mouse model of AD replicating numerous hallmarks of the disease. We performed both intracellular glucose imaging with a genetically encoded fluorescence resonance energy transfer (FRET)-based glucose biosensor and transcriptomic profiling of key molecular elements of glucose metabolism with single-cell multiplex RT-PCR (scRT-mPCR). We found that juvenile pyramidal cells exhibit active glycolysis and pentose phosphate pathway at rest that are respectively enhanced and impaired in 3xTg-AD mice without alteration of neuronal glucose uptake or transcriptional modification. Given the importance of glucose metabolism for neuronal survival, these early alterations could initiate or at least contribute to the later neuronal dysfunction of pyramidal cells in AD.

## Introduction

Alzheimer’s disease (AD) is a neurodegenerative disorder characterized by progressive cognitive impairment and memory loss. The underlying neuropathology includes brain deposition of amyloid plaques formed by misfolded amyloid-β (Aβ), neurofibrillary tangles and neuronal loss (Braak and Braak, 1991; Hardy and Selkoe, 2002). The visualization by 18-fluoro-2-deoxy-*D*-glucose positron emission tomography of a decreased cerebral glucose utilization occurring in specific brain regions is one of the earliest functional signs of AD (Small et al., 2000; Dubois et al., 2016). It aggravates with the disease progression and even predicts the neuropathological diagnosis of AD (Mosconi, 2005; Mosconi et al., 2010).

The most vulnerable brain regions to Aβ deposition corresponds to areas where glucose is physiologically used beside its role as a substrate for oxidative phosphorylation in spite of oxygen availability (Vaishnavi et al., 2010; Vlassenko et al., 2010). This non-oxidative glucose utilization is referred to as “aerobic glycolysis” and includes the glycolysis itself, the pentose phosphate shunt and the glycogen synthesis. Under physiological conditions, these glucose metabolic pathways are highly compartmentalized between neurons and glial cells, glycolysis, and glycogen synthesis occurring mainly in astrocytes (Allaman et al., 2011; Fernandez-Fernandez et al., 2012).

Deregulation of the neuronal metabolic program can be deleterious since hyperactive glycolysis and glycogen synthesis in neurons are pro-apoptotic (Vilchez et al., 2007; Herrero-Mendez et al., 2009). In addition, the pentose phosphate pathway promotes long-term neuronal survival (Vaughn and Deshmukh, 2008) and the non-oxidative use of glucose in the hexosamine biosynthetic pathway protects tau protein from hyperphosphorylation and restricts neurofibrillary tangles formation (Liu et al., 2009). Hence, an early alteration of neuronal glucose metabolism is likely to initiate or at least contribute to later neural dysfunctions and neurodegenerative processes characteristic of AD.

Although *in vivo* studies are considered as the reference standard to measure glucose metabolism (Sokoloff et al., 1977; Fox and Raichle, 1986; Fox et al., 1988), they generally lack the required resolution to investigate cellular processes and offer limited pharmacological investigations (Sotelo-Hitschfeld et al., 2015; Diaz-Garcia et al., 2017). Conversely, *in vitro* models provide an excellent spatial and temporal resolution but are largely based on culture systems derived from embryonic or neonate animals (Bittner C.X. et al., 2010; Surin et al., 2012; Tantama et al., 2013; Lerchundi et al., 2015). Given that brain metabolism undergoes substantial changes during embryonic and postnatal development (Bilger and Nehlig, 1992; Nehlig and Pereira de Vasconcelos, 1993; Nehlig, 1999; Surin et al., 2012) these *in vitro* models may not be appropriate to study age-related neurological disorders. The acute slice (*ex vivo*) models provide an intermediate approach preserving the cytoarchitecture of brain tissues also allowing cell-type specific investigations (Kasischke et al., 2004; Choi et al., 2012; Sotelo-Hitschfeld et al., 2015; Diaz-Garcia et al., 2017) but require careful design of experimental conditions (Iadecola and Nedergaard, 2007).

3xTg-AD mice is a model that replicates the neuropathological hallmarks of AD (Oddo et al., 2003; Carroll et al., 2010). In these mice, glucose metabolic impairments are already observed during embryogenesis (Yao et al., 2009) and progress during aging, thus preceding the apparition of Aβ deposition and tau hyperphosphorylation (Nicholson et al., 2010; Ding et al., 2013; Sancheti et al., 2014a,b; Sanguinetti et al., 2018). Since cerebral metabolism switches from ketone bodies to glucose utilization during the first postnatal weeks (Bilger and Nehlig, 1992; Nehlig and Pereira de Vasconcelos, 1993; Nehlig, 1999) this developmental stage might represent a critical step in the emergence of glucose metabolism alterations. Layer II and III pyramidal neurons already contribute to glucose supply via neurovascular interactions during this period (Cauli and Hamel, 2010; Lecrux et al., 2011; Lacroix et al., 2015) and have been reported to degenerate before Aβ deposition in 3xTg-AD mice (Bittner T. et al., 2010), suggesting an early involvement in the pathogenesis of AD. We therefore underwent our investigations at a presymptomatic juvenile stage corresponding to the developmental metabolic switch by comparing the metabolic program of supragranular pyramidal neurons of non-Tg and 3xTg-AD mice.

We used a genetically encoded fluorescence resonance energy transfer (FRET)-based glucose biosensor (Deuschle et al., 2005; Takanaga et al., 2008; Bittner C.X. et al., 2010) to investigate the uptake and fate of glucose at a cellular resolution and single-cell multiplex RT-PCR (scRT-mPCR) to determine the expression profiles of key elements of glucose metabolism of pyramidal cells. We report that both glycolysis and pentose phosphate pathways are active in juvenile pyramidal cells at rest. These fluxes are respectively enhanced and reduced in 3xTg-AD mice, without alteration of neuronal glucose uptake or transcriptional modification.

## Materials and Methods

### Animals

Homozygous 3xTg-AD (MMRRC #034830) generated and maintained on a mixed J29/C57BL6 background and wild type non-transgenic mice of the same background as well as C57BL6 background (11–19 postnatal days) of both genders were used for glucose imaging and patch-clamp recordings in acute slices. 3xTg-AD mice express the mutated gene PS1M146V (knock-in) and the mutated human genes APPSwe and tauP301L in the same locus, both under the control of the mouse Thy1.2 regulatory element and were routinely genotyped by PCR (Oddo et al., 2003). All animals were housed in a temperature-controlled (21–25°C) room under 12 h light:12 h dark conditions and were given food and water *ad libitum*. A maximum of five mice were housed per cage and single animal housing was avoided. All experimental procedures using animals were performed in strict accordance with French regulations (Code Rural R214/87 to R214/130) R214/126 and conformed to the ethical guidelines of both the European Economic Community (86/609/EEC) and the French National Charter on the ethics of animal experimentation. All protocols were approved by the Charles Darwin ethics committee and submitted to the French Ministry of Education and Research (Approval 2015 061011367540 APAFIS#573-2015061011367547 v1). The IBPS animal facility is accredited by the French authorities (A75-05-24).

### Subcloning and Viral Production

The glucose sensor FLII^12^Pglu-700μδ6 (Takanaga et al., 2008) was used in this study. The coding sequence of the sensor was subcloned into the viral vector pSinRep5 (Invitrogen). Sindbis virus were produced as previously described (Hepp et al., 2007; Hu et al., 2011). Recombinant pSinRep5 and helper plasmid pDH26S were transcribed *in vitro* into capped RNA using the Megascript SP6 kit (Ambion). Baby hamster kidney-21 (ATGC #CCL-10) cells were electroporated with sensor-containing RNA and helper RNA (2 × 10^7^ cells, 950 μF, 230 V) and incubated for 24 h at 37°C in 5% CO_2_ in Dulbecco’s modified Eagle Medium supplemented with 5% fetal calf serum before collecting cell supernatant containing the viruses. The virus titer (10^8^ infectious particles/ml) was determined after counting fluorescent baby hamster kidney cells infected using serial dilution of the stock virus.

### Slice Preparation

Mice were deeply anesthetized with isoflurane. After decapitation brains were quickly removed and placed into cold (∼4°C) oxygenated artificial cerebrospinal fluid (aCSF) containing (in mM): 126 NaCl, 2.5 KCl, 1.25 NaH_2_PO_4_, 2 CaCl_2_, 1 MgCl_2_, 26 NaHCO_3_, 10 glucose, 15 sucrose, and 1 kynurenic acid (Sigma). Coronal slices (300 μm thick) containing the barrel cortex were cut with a vibratome (VT1000S; Leica) and allowed to recover at room temperature for at least 1 h in aCSF saturated with O_2_/CO_2_ (95%/5%) as previously described (Karagiannis et al., 2009).

### Brain Slice Viral Infection

Brain slices were placed onto a millicell-CM membrane (Millipore) with culture medium (50% minimum essential medium, 50% Hank’s balanced salt sodium, 6.5 g/l glucose (∼36 mM), and 100 U/ml penicillin/100 μg/ml streptomycin; Invitrogen) as previously described (Drobac et al., 2010; Hu et al., 2011). Infection was performed by adding ∼5 × 10^5^ particles per slice. Slices were incubated overnight at 35°C in 5% CO_2_. The next morning, brain slices were equilibrated for 1 h in aCSF containing (in mM): 126 NaCl, 2.5 KCl, 1.25 NaH_2_PO_4_, 2 CaCl_2_, 1 MgCl_2_, 26 NaHCO_3_, 2.5 glucose, and 22.5 sucrose to reduce glucose concentration to a physiological level (Silver and Erecinska, 1994). Slices were then placed into the recording chamber, heated at ∼30°C and continuously perfused at 1–2 ml/min.

### Double-Immunofluorescence Labeling

Following viral transduction, slices were fixed overnight at 4°C in 0.1 M phosphate-buffer containing 4% formaldehyde. Then, slices were rinsed with phosphate-buffer saline (PBS), permeabilized with PBS/gelatin 0.2%/Triton 0.25%, and incubated overnight at 4°C with rabbit anti-Satb2 (1:1000, ab34735, Abcam; Lee et al., 2010) and chicken anti-GFP (1:1000, GFP-1020, Aves Labs; Tricoire et al., 2010). After washing in PBS, the respective immunoreactions were visualized with the following secondary antibodies: goat-anti-rabbit AlexaFluor 555 (1:1000, A-21430, Thermo Fisher Scientific) and goat-anti chicken AlexaFluor 488 (1:1000, A-11039, Thermo Fisher Scientific) incubated 1 h at room temperature. Sections were mounted with fluoromount-G (Southern Biotech) on slides for visualization. Images of immunostained material were acquired using a Leica TCS SP5 AOBS inverted confocal microscope with a 40× objective (40× HCX P APO CS NA 1.25–0.75/Oil) and LAS AF software (Leica Microsystems). Cell counting was performed using Image Pro Analyzer 7.0.0.951 (MediaCybernetics).

### FRET Imaging

Recordings were made from visually identified pyramidal cells in layer II and III of the mouse somatosensory cortex. Wide-field fluorescent images were obtained using a double port upright microscope (BX51WI, WI-DPMC Olympus) with a 40x objective (LUMPlan FL N 40×/0.80 W) and a digital camera (CoolSnap HQ2, Roper Scientific or Orca Flash 4.0, Hamamatsu) attached on the front port of the microscope. The glucose sensor FLII^12^Pglu-700μδ6 was excited at 400 nm with a light emitting device (LED; CoolLED, Precise Excite) using Imaging Workbench 6.0.25 software (INDEC BioSystems) and excitation (HC 438/24, Semrock) and dichroic filters (HC BS 458, Semrock). Double fluorescence images were collected every 15 s by alternating the fluorescence emission filters for the CFP (HC 483/32 Semrock) and the YFP (HC 542/27, Semrock) using a filter wheel (Lambda 10B, Sutter Instruments). In parallel, infrared transmitted light images of slices were continuously monitored on the back-port of the microscope using an infrared collimated LED as a light source (780 nm, Thorlabs), Dodt gradient contrast optics (DGC; Luigs and Neumann; Dodt and Zieglgansberger, 1998), a customized beam splitter (725 DCSPXR, Semrock) and an analogic CCD camera (XC ST-70 CE, Sony). The focal plane was maintained constant on-line using infrared DGC images of cells as anatomical landmarks (Lacroix et al., 2015). To compensate for potential *x*–*y* drifts all images were realigned off-line using the “StackReg” plug-in (Thevenaz et al., 1998) of ImageJ 1.48 software. Fluorescence ratios were calculated by dividing the registered YFP images by the registered CFP images using FIJI^1^. On average 8.0 ± 0.3 transduced neurons were visualized in the field of view of the CoolSnap HQ2 with no statistical difference between 3xTg-AD and non-Tg mice (*U*(29,30) = 390, *p* = 0.5027). To determine regions of interests (ROIs) corresponding to the cytosolic part of the soma, nuclei were manually delineated and excluded from somatic ROIs. The mean ratio was measured at each time point using the same ROIs. The experiments lasted more than 30 min, leading to a small drift in the ratio baseline (**Figure 2B**). The drift was corrected by a linear curve fitting (John et al., 2008) using Clampfit 10.4 software (MDS). Variations of fluorescence ratio were expressed as the ratio (R–R0)/R0, where R corresponds to the fluorescence ratio in the ROI at a given time point, and R0 corresponds to the mean fluorescence ratio in the same ROI during the 10 min control baseline prior to changes in extracellular glucose concentration or application of drugs. Relative ratio traces were smoothed with a sliding five-point median filter to eliminate sharp artifacts resulting from transient loss of focus.

### Whole-Cell Recordings in Acute Slices

Patch pipettes (4–6 MΩ) pulled from borosilicate glass were filled with 8 μl of RNAse free internal solution containing in (mM): 144 K-gluconate, 3 MgCl_2_, 0.5 EGTA, 10 HEPES, pH 7.2 (285/295 mOsm). Whole-cell recordings were performed at 27.0 ± 0.5°C using a patch-clamp amplifier (Axopatch 200B, MDS). Data were filtered at 5–10 kHz and digitized at 50 kHz using an acquisition board (Digidata 1440, MDS) attached to a personal computer running pCLAMP 10.2 software package (MDS). Membrane potential values were corrected for liquid junction potential (-15.6 mV). Resting membrane potential of neurons was measured just after passing in whole-cell configuration. Only neurons with a resting membrane potential more negative than -60 mV were analyzed further. A total of 32 electrophysiological properties chosen to describe the electrophysiological diversity of cortical neurons (Ascoli et al., 2008) were determined as previously described (Karagiannis et al., 2009).

### Cytoplasm Harvesting and scRT-PCR

At the end of the whole-cell recording, lasting <15 min, the cytoplasmic content was aspirated in the recording pipette. The pipette’s content was expelled into a test tube and RT was performed in a final volume of 10 μl, as described previously (Lambolez et al., 1992). The scRT-PCR protocol was designed to probe simultaneously the expression of neuronal markers and key molecular elements of glucose metabolism. Two-steps amplification was performed essentially as described (Cauli et al., 1997; Devienne et al., 2018). Briefly, cDNAs present in the 10 μl reverse transcription reaction were first amplified simultaneously using all external primer pairs listed in **Table 1**. Taq polymerase (2.5 U; Qiagen) and 20 pmol of each primer were added to the buffer supplied by the manufacturer (final volume, 100 μl), and 20 cycles (94°C, 30 s; 60°C, 30 s; and 72°C, 35 s) of PCR were run. Second rounds of PCR were performed using 1 μl of the first PCR product as a template. In this second round, each cDNA was amplified individually using its specific nested primer pair (**Table 1**) by performing 35 PCR cycles (as described above). A ten μl of each individual PCR product were run on a 2% agarose gel stained with ethidium bromide using ΦX174 digested by *Hae*III as a molecular weight marker.

**Table 1:** PCR primers.

Genes Accession #	First PCR primers	Size (bp)	Second PCR nested primers	Size (bp)
vGluT1	Sense, -113: GGCTCCTTTTTCTGGGGCTAC	259	Sense, -54: ATTCGCAGCCAACAGGGTCT	153
NM_182993	Antisense, 126: CCAGCCGACTCCGTTCTAAG		Antisense, 79: TGGCAAGCAGGGTATGTGAC	
GAD65	Sense, 99: CCAAAAGTTCACGGGCGG	375	Sense, 219: CACCTGCGACCAAAAACCCT	248
NM_008078	Antisense, 454: TCCTCCAGATTTTGCGGTTG		Antisense, 447: GATTTTGCGGTTGGTCTGCC	
GAD67	Sense, 529: TACGGGGTTCGCACAGGTC	598	Sense, 801: CCCAGAAGTGAAGACAAAAGGC	255
NM_008077	Antisense, 1,109: CCCAGGCAGCATCCACAT		Antisense, 1,034: AATGCTCCGTAAACAGTCGTGC	
GluT1	Sense, 5: ATCCCAGCAGCAAGAAGGTGA	333	Sense, 88: ACTGGTGTCATCAACGCCCC	164
NM_011400	Antisense, 317: AGAAGCCCATAAGCACAGCAG		Antisense, 230: CCGACAGAGAAGGAACCAATCA	
GluT3	Sense, 1: ATGGGGACAACGAAGGTGAC	358	Sense, 23: CATCTCTGGTGTTCGCCGT	310
NM_011401	Antisense, 336: GCATTTCAACAGACTCCGCTATC		Antisense, 314: GCGAATCCCATAAGGCAGC	
HK1	Sense, 879: CGAGAAGATGGTGAGCGGC	472	Sense, 986: TCACGAGGGGCAAGTTCACC	330
NM_001146100	Antisense, 1,333: GCCACTGCCACTCTCCGA		Antisense, 1,298: CCGAGTCAGGCACCAGGC	
Pfkfb3	Sense, 211: GTGGGAGAGTATCGGCGTGA	494	Sense, 255: CAACTTCTTCCGCCCTGACA	365
NM_001177752	Antisense, 685: ATTCGGCTCTGGATGTGGTC		Antisense, 596: CACATTTATCAGGGTCAAGAGGCT	
PFK1m	Sense, 49: GCCATCGCCGTGTTGACC	247	Sense, 106: GCTGTGGTCCGAGTTGGTATCT	160
NM_001163487	Antisense, 276: GTCGTCCTTCTCGCTCTCGG		Antisense, 246: ATCGGGCACTTCCAATCACT	
PFK1l	Sense, 180: AGGAGGCGAGAACATCAAGC	408	Sense, 304: GCCTACAATCTGCTCCAACACG	169
NM_008826	Antisense, 568: GCAGTGGTAGTGATGGCGTC		Antisense, 451: TGGTCAAGTGTGCGTAGTTCTG	
PFK1p	Sense, 409: CGAAAGGAGTGGAGCGGA	306	Sense, 438: GCTGGCTCGGAATGGTGAT	219
NM_019703	Antisense, 696: AAGGAACACCCAGTCGGCA		Antisense, 637: TGTCTCCCCATCACCTCCAG	
G6PDx	Sense, 206: GCTATGCCCGCTCACGAC	303	Sense, 491: CCACGATGATGCGGTTCC	143
NM_008062	Antisense, 232: GACGACATCCGAAAGCAGAGTG		Antisense, 353: ATGTGGCTGTTGAGGTGCTTAT	
Gys1	Sense, 3: GCCTCTCAGCCGCAGTCT	313	Sense, 66: CGACCCCGAGAACGCAGT	157
NM_030678	Antisense, 294: ACGCCCAAAATACACCTTACAA		Antisense, 205: CCTCACACCCTGCTCCGT	
PygB	Sense, 108: GCATTTCACGCTGGTCAAGG	292	Sense, 156: CTTCTTCGCTCTGGCACACA	110
NM_153781	Antisense, 380: CCCAAGACCAGCATCCTCCT		Antisense, 244: CCAGGGAAAGGTAATAGATGCG	

*Position 1, first base of the start codon.*

### Drugs

Changes in extracellular glucose concentration were compensated by changes in sucrose concentration to maintain the osmolarity of the aCSF constant as previously described (Miki et al., 2001). Iodoacetic acid (IAA) and 6-aminonicotinamide (6-AN) were purchased from Sigma-Aldrich.

### Statistical Analyses

Statistical analyses were performed with Statistica 6 (Statsoft). All values are expressed as mean ± SEM. Statistical significance of FRET ratio changes and electrophysiological properties of pyramidal cells of 3xTg-AD and non-Tg mice was determined using the Mann–Withney *U* test. Comparison of the occurrence of the genes expressed by pyramidal cells of the two mice lines was determined using Fisher’s exact test. Statistical significance on all figures uses the following convention: ^∗^*p* < 0.05, ^∗∗^*p* < 0.01, and ^∗∗∗^*p* < 0.001.

## Results

### Glucose Uptake

**FIGURE 1:**
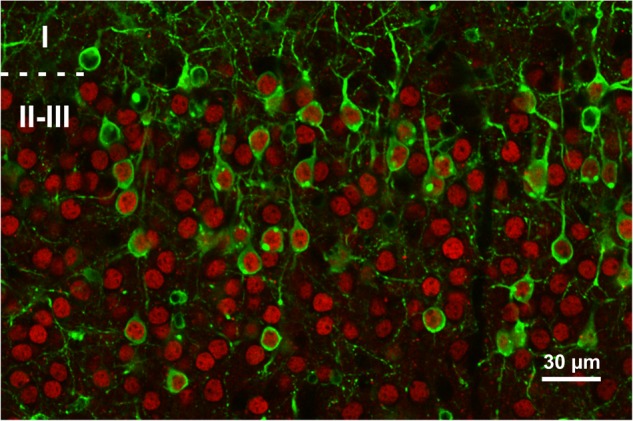
Expression of the FRET glucose sensor in neocortical slices. Representative single plane confocal image of a slice expressing the FLII12Pglu-700 μδ6 biosensor following sindbis viral transduction. Cells expressing the FRET glucose sensor are identified by their GFP fluorescence (*green*). Pyramidal cells are immunolabeled for the Satb2 transcription factor (*red*). *Dashed line* represents layer I and II border. Note that most layer II and III transduced cells are stab2 positive.

**FIGURE 2:**
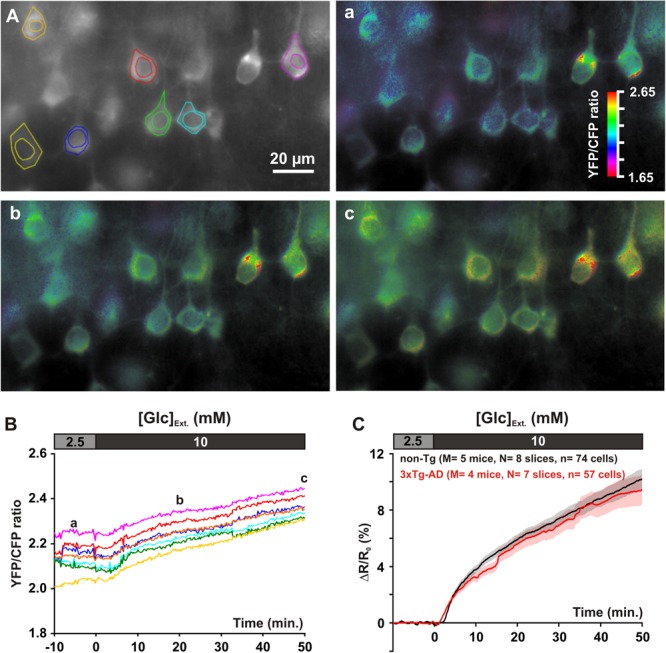
Glucose uptake in layer II and III pyramidal cells. **(A)** The gray scale image shows the YFP fluorescence. Pseudocolor images show the YFP/CFP ratio value [coded by pixel hue, see scale bar in **(a)**] and the fluorescence intensity (coded by pixel intensity) at different time points before **(a)** and after **(b,c)** superfusion of 10 mM glucose. **(B)** Traces show average YFP/CFP ratio measure at the cytosolic part of the soma of individual pyramidal cells delineated in **(A)**. **(a–c)** indicate time points corresponding to the pseudocolor images shown in **(A)**. **(C)** Mean relative YFP/CFP ratio changes in pyramidal cells of non-Tg (*black trace*) and 3xTg-AD mice (*red trace*) induced by superfusion of 10 mM glucose. Traces show mean (*solid lines*) ± standard errors of the mean (*color shades*). Note the slowly developing increase in mean relative ratio during 10 mM glucose superfusion and the similar temporal profiles between non-Tg and 3xTg-AD mice.

We used a recombinant Sindbis virus (see Materials and Methods) to express the FRET glucose sensor FLII^12^Pglu-700μδ6 (Takanaga et al., 2008) in neurons of neocortical slices. Expression of the biosensor was preferentially observed in pyramidal shaped cells of layers II and III (**Figures 1, 2A**), consistent with previous reports (Drobac et al., 2010; Hu et al., 2011). The sensor was observed both in somata and in proximal dendrites, but virtually absent from nuclei (**Figures 1, 2A**). The pyramidal identity of transduced cells was next examined by double immunofluorescence for GFP and Satb2, a pyramidal cells transcription factor (Britanova et al., 2008; Lee et al., 2010; Lacroix et al., 2015). A large majority of transduced cells displayed a nucleus positive for Satb2 (75.1 ± 3.1%, *n* = 415 GFP-expressing cells from three different mice) confirming the pyramidal tropism of the Sindbis virus (Chen et al., 2000; Lendvai et al., 2000; Furuta et al., 2001; Drobac et al., 2010). Optical recordings were then performed in layer II and III by measuring the YFP/CFP emission ratio of the biosensor in the cytosol.

The resting intracellular glucose concentration is determined by glucose transport (uptake and efflux) and metabolism (Bittner C.X. et al., 2010; Hou et al., 2011). We first evaluated the capability of supragranular neuron to take-up glucose by monitoring dynamic changes in intracellular glucose during an increase in extracellular glucose concentration (John et al., 2008; Takanaga et al., 2008; Hou et al., 2011). Increasing extracellular glucose from 2.5 mM, a physiologically relevant glucose concentration (Silver and Erecinska, 1994), to 10 mM, a glucose concentration nearly saturating the sensor (Takanaga et al., 2008) and classically used in brain slices experiments, lead to a slowly developing increase in fluorescence emission ratio (**Figures 2B,C**). This confirms that pyramidal neurons from juvenile mice of both genotypes have the capability to take up glucose (Diaz-Garcia et al., 2017).

During application of 10 mM glucose the relative increase in fluorescence ratio was 3.8 ± 0.3% in non-Tg neurons vs. 3.2 ± 0.3% in 3xTg-AD neurons after 10 min [*U*(74,57) = 1,787, *p* = 0.1359], 5.9 ± 0.4% vs. 5.6 ± 0.6% [*U*(74,57) = 1,844, *p* = 0.2203] after 20 min, 7.4 ± 0.5% vs. 6.9 ± 0.7% [*U*(74,57) = 1,822, *p* = 0.1841] after 30 min, 8.8 ± 0.6% vs. 8.4 ± 0.9% [*U*(74,57) = 1,843, *p* = 0.2185] after 40 min and 10.2 ± 0.7% vs. 9.4 ± 1.1% after [*U*(74,57) = 1,785, *p* = 0.1335] after 50 min. These results revealed that the glucose accumulations were very similar between pyramidal cells of juvenile non-Tg and 3xTg-AD mice (**Figure 2C**). Since in slices we cannot estimate the resting intracellular glucose concentration, we next thought to determine whether the elimination rate of glucose was similar. To do so, we reduced extracellular glucose concentration close to 0 (Bittner C.X. et al., 2010).

### Glucose Metabolic Activity

**FIGURE 3:**
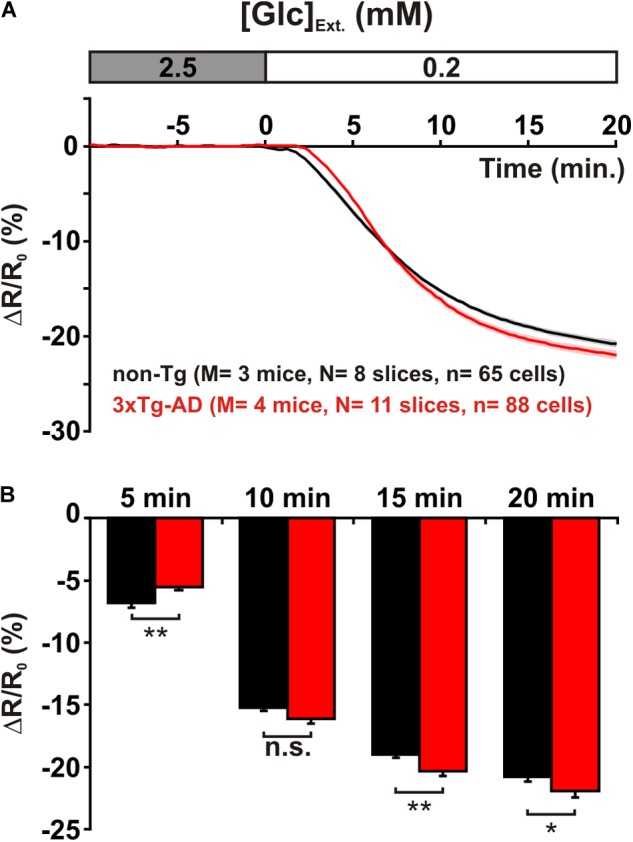
Metabolic activity of layer II and III pyramidal cells. **(A)** Mean relative YFP/CFP ratio changes in pyramidal cells of non-Tg (*black trace*) and 3xTg-AD mice (*red trace*) induced by glucose (Glc) restriction (0.2 mM). Traces show mean (*solid lines*) ± standard errors of the mean (*color shades*). Note the slowly developing decrease in mean relative ratio during 0.2 mM Glucose superfusion. Note also the difference in the temporal profiles between non-Tg and 3xTg-AD mice. **(B)** Histograms show the metabolic activity (decrease in relative YFP/CFP ratio) at different time of glucose restriction. After 5 min of glucose restriction pyramidal cells of non-Tg mice exhibit a higher metabolic activity than those of 3xTg-AD mice. In contrast, during longer glucose restriction pyramidal cells of 3xTg-AD mice display a higher metabolic activity. n.s. not statistically significant.

Decreasing extracellular glucose from 2.5 to 0.2 mM led to a marked drop in relative fluorescence ratio (**Figure 3A**). This indicates that pyramidal cells metabolize glucose under resting condition as recently reported (Diaz-Garcia et al., 2017). The slopes of intracellular glucose decrease, measured between 2 and 7 min after glucose restriction, were not statistically different between non-Tg and 3xTg-AD pyramidal cells [-2.0 ± 0.0% min^-1^ vs. -2.2 ± 0.1% min^-1^, *U*(65,88) = 2,666, *p* = 0.4740]. This indicates that the elimination rates of glucose, which depend on both efflux and metabolism, were similar. Taken together, the similar accumulation and elimination rates we observed (**Figures 2, 3**) revealed that the capabilities of glucose transport are comparable between non-Tg and 3xTg-AD pyramidal cells.

Nonetheless, scrutinization of the dynamics of intracellular glucose revealed subtle differences between the metabolic activity of pyramidal cells of juvenile non-Tg and 3xTg-AD mice (**Figure 3A**). After 5 min of extracellular glucose restriction, the drop was stronger in non-Tg than in 3xTg-AD mice [-6.8 ± 0.4% vs. -5.5 ± 0.3%, *U*(65,88) = 2,087, *p* = 0.0043] indicating a higher metabolic activity in non-Tg mice at an early stage of glucose restriction (**Figure 3**). However it did not differ after 10 min of glucose restriction [-15.2 ± 0.3% vs. -16.1 ± 0.4%, *U*(65,88) = 2,444, *p* = 0.1247, **Figure 3B**]. Interestingly, longer glucose restrictions disclosed a higher elimination rate of glucose in 3xTg-AD pyramidal cells (**Figure 3**), which was evident after 15 min [-19.0 ± 0.3% vs. -20.3 ± 0.4%, *U*(65,88) = 2,029, *p* = 0.0022] and persisted after 20 min [-20.8 ± 0.4% vs. -21.9 ± 0.5%, *U*(65,88) = 2,237, *p* = 0.0215, **Figure 3B**]. The lower and higher glucose elimination rates, respectively observed at early and late stages of glucose depletion in 3xTg-AD pyramidal cells, may suggest that at least two glucose pathways with different kinetics are altered. The similar elimination rates we observed indicate that these alterations roughly counterbalance each other.

**FIGURE 4:**
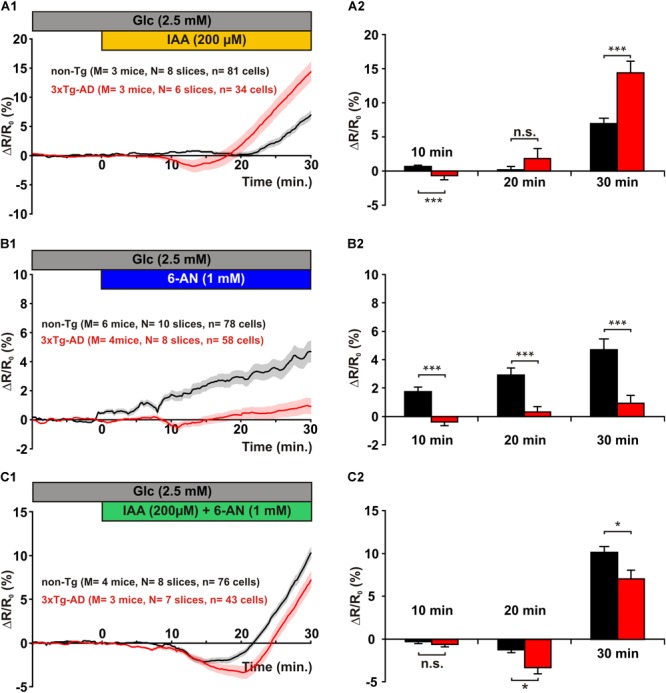
Glucose metabolic pathways in layer II and III pyramidal cells. *Left* panels show the mean relative YFP/CFP ratio changes in pyramidal cells of non-Tg (*black traces*) and 3xTg-AD mice (*red traces*) induced by glycolysis inhibition with IAA **(A1)**, pentose phosphate pathway inhibition with 6-AN **(B1)**, and combined inhibition with both IAA and 6-AN **(C1)**. Traces show mean (*solid lines*) ± standard errors of the mean (*color shades*). Histograms show the metabolic activity (decrease in relative YFP/CFP ratio) at different time of glycolysis inhibition **(A2)**, pentose phosphate pathway inhibition **(B2)**, and combined inhibition **(C2)**. n.s. not statistically significant. **(A1)** Note the delayed increase in mean relative ratio in non-Tg pyramidal cells during IAA application. Note also the early decrease followed by a late increase in pyramidal cells of 3xTg-AD mice. **(A2)** After 10 min of IAA application pyramidal cells of 3xTg-AD mice develop a transient increase in metabolic activity. Longer application reveals a larger impact of glycolysis inhibition in pyramidal cells of 3xTg-AD mice than in pyramidal cells of non-Tg mice. **(B1)** Note the very low increase in mean relative ratio during 6-AN application in pyramidal cells of 3xTg-AD mice. **(B2)** The mean increase in relative ratio is higher in pyramidal cells of non-Tg mice at 10, 20, and 30 min of 6-AN superfusion. **(C1)** Note the transient decrease in mean relative ratio during combined IAA and 6-AN application more pronounced in pyramidal cells of 3xTg-AD mice than in non-Tg neurons. **(C2)** After 20 min of combined inhibition pyramidal cells of 3xTg-AD mice exhibit a marked transient increase in metabolic activity. Longer application reveals an accumulation of glucose smaller in pyramidal cells from 3xTg-AD than from non-Tg mice.

To evaluate the relative contribution of different glucose pathways to the metabolic activity of pyramidal cells, we monitored glucose accumulation induced by different metabolic inhibitors. We first inhibited glycolysis using IAA, a widely used glyceraldehyde-3-phosphate dehydrogenase (G3PDH) inhibitor that does not interfere with the FRET glucose sensors (John et al., 2008; Bittner C.X. et al., 2010). We used 200 μM IAA, a concentration that efficiently inhibits glycolysis in brain slices (Yang et al., 1999; Gordon et al., 2008) with modest impact on the pentose phosphate pathway or lactate dehydrogenase activity (Sabri and Ochs, 1971; Schmidt and Dringen, 2009). Bath application of IAA induced a delayed increase in relative fluorescence ratio in non-Tg pyramidal cells (**Figure 4A1**), indicative of glucose accumulation and consistent with glycolytic activity in cortical neurons under resting condition (Bittner C.X. et al., 2010; Diaz-Garcia et al., 2017). Surprisingly, in pyramidal cells of 3xTg-AD mice IAA led to a biphasic response consisting in a transient decrease in intracellular glucose followed by a latter increase (**Figure 4A1**). The transient decrease, absent in pyramidal cells of non-Tg mice, was observable after 10 min of IAA application [0.7 ± 0.2% vs. -0.7 ± 0.6%, *U*(81,34) = 569, *p* = 2.480×10^-7^, **Figure 4A2**] and lasted for about 10 min. At that time of glycolysis inhibition, the intracellular glucose dynamics of non-Tg and 3xTg-AD pyramidal cells crossed (**Figure 4A1**) and did not differ significantly [0.2 ± 0.5% vs. 1.8 ± 1.4%, *U*(81,34) = 1,341, *p* = 0.8286, **Figure 4A2**]. This transient decrease in glucose level observed under glycolysis inhibition suggests that another glucose-consuming metabolic pathway was stimulated or at least disclosed. A longer exposure to IAA revealed a higher intracellular glucose increase in 3xTg-AD neurons [7.0 ± 0.8% vs. 14.4 ± 1.7%, *U*(81,34) = 636, *p* = 2.715×10^-6^ at 30 min]. These observations suggest that the glycolysis rate is higher in 3xTg-AD than in non-Tg pyramidal cells.

We next evaluated the importance of the pentose phosphate shunt in the metabolism of pyramidal cells and thought to determine whether it is altered in 3xTg-AD pyramidal cells. Applying 6-AN induced a slowly developing increase in intracellular glucose concentration in non-Tg pyramidal cells (**Figure 4B1**). After 30 min the amplitude of this increase reached a level comparable to the one observed under glycolysis inhibition (**Figures 4A1, B1**), indicating that both pathways contribute to the resting glucose metabolism of pyramidal cells.

In contrast, in 3xTg-AD neurons 6-AN induced a slight and transient decrease in intracellular glucose observable after 10 min [1.7 ± 0.3% vs. -0.4 ± 0.3%, *U*(78,58) = 1,120, *p* = 2.393×10^-7^] followed by a very mild increase at 20 min [2.9 ± 0.5% vs. 0.3 ± 0.4%, *U*(78,58) = 1,365, *p* = 6.195×10^-5^] and 30 min of exposure [4.7 ± 0.8% vs. 0.9 ± 0.6%, *U*(78,58) = 1,389, *p* = 9.861×10^-5^, **Figure 4B2**], respectively. These results reveal that the pentose phosphate pathway is dramatically hampered in 3xTg-AD neurons.

In order to investigate further the metabolic pathway disinhibited in 3xTg-AD neurons during glycolysis inhibition, we simultaneously applied IAA and 6-AN to inhibit both glycolysis and the pentose phosphate pathway. Such a combined inhibition induced a biphasic response in both non-Tg and 3xTg-AD pyramidal cells consisting in a small and transient decrease in intracellular glucose followed by a later increase (**Figure 4C1**). However, the initial dip in intracellular glucose was much stronger and longer lasting in 3xTg-AD neurons (**Figure 4C1**). Indeed, it was comparable after 10 min of combined inhibition [-0.3 ± 0.2% vs. -0.6 ± 0.3%, *U*(76,43) = 1,479, *p* = 0.3943] and was particularly marked after 20 min [-1.3 ± 0.4% vs. -3.3 ± 0.7%, *U*(76,43) = 1,228, *p* = 0.0245, **Figure 4C2**]. Since this transient glucose consumption in 3xTg-AD neurons was observed under both glycolysis (**Figure 4A1**) and combined inhibition (**Figure 4C1**) and barely during inhibition of the pentose phosphate pathway alone (**Figure 4B1**), it indicates that glycolysis inhibition is required to disclose the initial dip. It also suggests that the pentose phosphate pathway does not chiefly underlay this transient glucose consumption. A longer combined inhibition resulted in an intracellular glucose increase that was higher in non-Tg than in 3xTg-AD pyramidal cells [10.2 ± 0.7% vs. 7.1 ± 1.0%, *U*(76,43) = 1,267, *p* = 0.0423, **Figures 4C1, C2**]. These observations suggest that the lower pentose phosphate activity observed in 3xTg-AD (**Figure 4A**) neurons is partially compensated by a higher glycolytic activity (**Figures 4B,C**).

### Molecular Characterization of Layer II and III Pyramidal Cells

**FIGURE 5:**
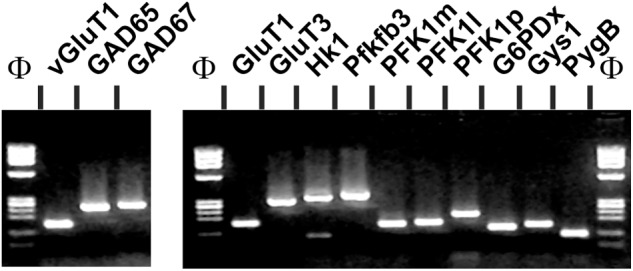
Sensitivity of the RT-mPCR. Total whole brain RNAs (1 ng) were subjected to a RT-PCR protocol. The PCR products were resolved in separate lanes by agarose gel electrophoresis in parallel with Φ×174 digested by *Hae*III as molecular marker and stained with ethidium bromide. The amplified fragments correspond to neuronal markers (*left*), glucose transporters and key enzymes of glucose metabolic pathways (*right*).

To determine the main genes of glucose metabolism underlying the metabolic program of pyramidal cells and to evaluate whether transcriptomic modifications could mediate the alterations observed in 3xTg-AD mice, we analyzed the expression profiles of these genes in non-Tg and 3xTg-AD pyramidal cells by single cell RT-multiplex PCR (scRT-mPCR). The protocol was designed to probe the expression of vGluT1 and GAD65 and 67, taken as glutamatergic and GABAergic markers, respectively. The key genes of glucose metabolism included two glucose transporters GluT1 and GluT3, the hexokinase isoform HK1, the main brain phosphofructokinase-2 isoform; Pfkfb3, the muscle, liver and platelet isoforms of phosphofructokinase-1 (PFK1m, l and p), the rate limiting enzyme of the pentose phosphate pathway (glucose-6-phosphate dehydrogenase, G6PDx), as well as glycogen synthase and phosphorylase isoforms Gys1 and PygB. The reliability of the scRT-mPCR was tested on 1 ng of total RNA purified from mouse whole brain. As expected, all PCR generated fragments had the sizes predicted by their mRNA sequences (**Figure 5** and **Table 1**) confirming the sensitivity of the amplification procedure (Cauli et al., 1997).

**FIGURE 6:**
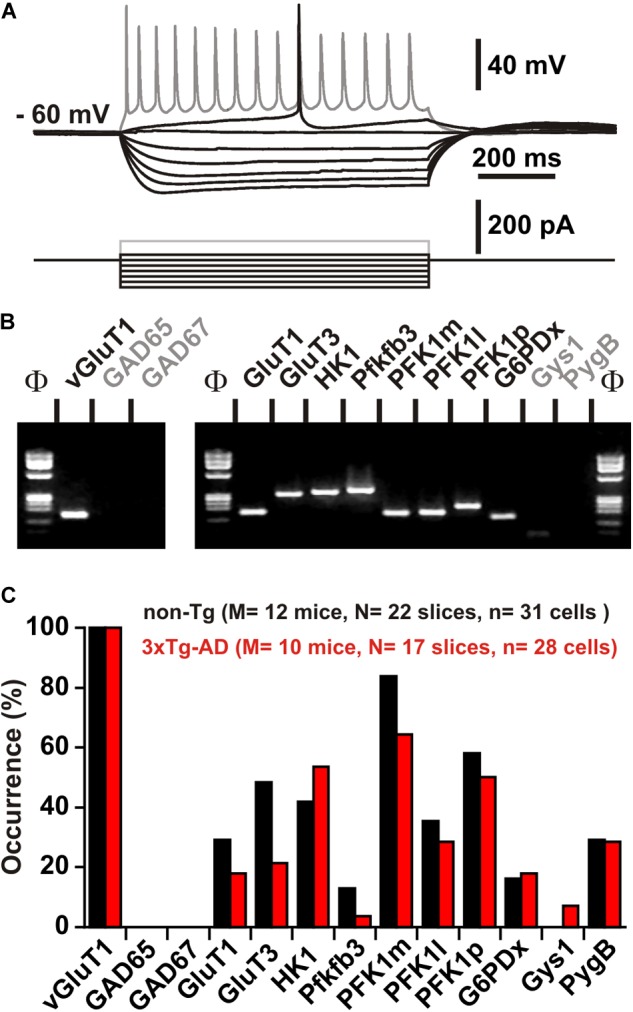
Characterization of key molecular elements of glucose metabolism in layer II and III pyramidal cells. **(A)** Voltage responses (*upper traces*) induced by injection of current pulses (*bottom traces*) in a non-Tg layer II and III pyramidal cell. In response to just-above-threshold current pulse, this neuron fired an action potential with a long lasting biphasic AHP (*middle trace*). Near saturation it showed the typical firing of a regular spiking neuron with marked frequency adaptation and spike amplitude accommodation (*shaded trace*). **(B)** The pyramidal cell shown in **(A)** expressed vGluT1, GluT1, GluT3, HK1, pfkfb3, PFK1m, PFK1l, PFK1p, and G6PDx. **(C)** Histograms showing the expression profiles of key molecular elements of glucose metabolism in layer II and III pyramidal cells of non-Tg (*black*) and 3xTg-AD mice (*red*). No statistically significant difference in the occurrence of genes was observed. Note the very low occurrence of pfkfb3 and Gys1 (glycogen synthase) in pyramidal neurons.

Layer II and III pyramidal cells were first visually identified on the basis of the triangular shape of their soma and by the presence of a prominent apical dendrite. Pyramidal cells typically fired long duration action potentials with a pronounced frequency adaptation (**Figure 6A** and **Tables 2–5**) characteristic of Regular Spiking neurons (McCormick et al., 1985; Cauli et al., 1997; Karagiannis et al., 2009; Lacroix et al., 2015). We did not observe any difference between the electrophysiological properties of non-Tg and 3xTg-AD juvenile pyramidal cells (**Tables 2–5**), consistent with previous reports (Yamamoto et al., 2011). Their glutamatergic phenotype was further confirmed by the detection of vGluT1 mRNAs and the absence of GAD65 and GAD67 transcripts (**Figures 6B,C**).

**Table 2:** Subthreshold properties of pyramidal cells.

	Non-Tg (*n* = 31)	3xTg-AD (*n* = 28)
Resting potential (mV)	–74.2 ± 1.6	–74.6 ± 1.4
	*U*(31,28) = 403	*p* = 0.6458
	n.s.	
Input resistance (MΩ)	541 ± 50	485 ± 42
	*U*(31,28) = 388	*p* = 0.4926
	n.s.	
Time constant (ms)	46.1 ± 3.0	46.4 ± 2.8
	*U*(31,28) = 415	*p* = 0.7805
	n.s.	
Membrane capacitance (pF)	96.2 ± 6.1	103.9 ± 6.3
	*U*(31,28) = 364	*p* = 0.2938
	n.s.	
Sag Index (%)	10.8 ± 1.2	11.8 ± 1.4
	*U*(31,28) = 389	*p* = 0.5022
	n.s.	

*n, number of cells; n.s., not statistically significant.*

*Mann–Whitney *U* tests and corresponding exact *p* values.*

**Table 3:** Just above threshold properties of pyramidal cells.

	Non-Tg (n = 31)	3xTg-AD (n = 28)
Rheobase (pA)	17.2 ± 4.4	18.1 ± 6.9
	*U*(31,28) = 426	*p* = 0.9101
	n.s.	
First spike latency (ms)	274.0 ± 24.4	248.9 ± 23.2
	*U*(31,28) = 376	*p* = 0.3855
	n.s.	
Adaptation (Hz/s)	–1.7 ± 1.2	–0.6 ± 0.4
	*U*(31,28) = 421	*p* = 0.8506
	n.s.	
Minimal steady state frequency (Hz)	5.2 ± 0.4	4.5 ± 0.3
	*U*(31,28) = 389	*p* = 0.5022
	n.s.	

*n, number of cells; n.s., not statistically significant.*

*Mann–Whitney *U* tests and corresponding exact *p* values.*

**Table 4:** Firing properties of pyramidal cells.

	Non-Tg (*n* = 31)	3xTg-AD (*n* = 28)
Amplitude accommodation (mV)	5.6 ± 1.2	6.8 ± 1.0
	*U*(31,28) = 321	*p* = 0.0878
	n.s.	
Amplitude of early adaptation (Hz)	42.1 ± 4.1	50.2 ± 4.2
	*U*(31,28) = 334	*p* = 0.1317
	n.s.	
Time constant of early adaptation (ms)	24.2 ± 1.5	22.7 ± 1.5
	*U*(31,28) = 372	*p* = 0.3532
	n.s.	
Maximal steady-state frequency (Hz)	29.3 ± 1.2	31.8 ± 1.6
	*U*(31,28) = 333	*p* = 0.1278
	n.s.	
Late adaptation (Hz/s)	–12.8 ± 0.8	–12.2 ± 0.7
	*U*(31,28) = 412	*p* = 0.7460
	n.s.	

*n, number of cells; n.s., not statistically significant.*

*Mann–Whitney *U* tests and corresponding exact *p* values.*

**Table 5:** Action potential waveforms of pyramidal cells.

	Non-Tg (*n* = 31)	3xTg-AD (*n* = 28)
1st spike amplitude (mV)	88.3 ± 1.7	88.7 ± 1.9
	*U*(31,28) = 398	*p* = 0.5926
	n.s.	
2nd spike amplitude (mV)	85.8 ± 1.7	86.4 ± 2.0
	*U*(31,28) = 391	*p* = 0.5216
	n.s.	
1st spike duration (ms)	1.7 ± 0.0	1.7 ± 0.1
	*U*(31,28) = 413	*p* = 0.7574
	n.s.	
2nd spike duration (ms)	1.8 ± 0.0	1.8 ± 0.1
	*U*(31,28) = 390	*p* = 0.5119
	n.s.	
Amplitude reduction (%)	2.9 ± 0.4	2.7 ± 0.4
	*U*(31,28) = 405	*p* = 0.6676
	n.s.	
Duration increase (%)	6.5 ± 0.7	5.7 ± 0.9
	*U*(31,28) = 353.5	*p* = 0.2236
	n.s.	
1st spike, 1st component AHP (mV)	–9.8 ± 0.7	–9.8 ± 0.5
	*U*(31,28) = 398.5	*p* = 0.5926
	n.s.	
1st spike, 1st AHP component latency (ms)	8.4 ± 0.4	9.3 ± 0.7
	U(31,28) = 431	*p* = 0.9700
	n.s.	
1st spike, 2nd component AHP (mV)	–15.7 ± 0.6	–15.4 ± 0.4
	U(31,28) = 361	*p* = 0.2733
	n.s.	
1st spike, 2nd AHP component latency (ms)	47.3 ± 2.2	44.1 ± 2.3
	U(31,28) = 364	*p* = 0.2938
	n.s.	
1st spike, ADP (mV)	0.0 ± 0.0	0.1 ± 0.0
	U(31,28) = 430	*p* = 0.9580
	n.s.	
1st spike, ADP latency (ms)	0.5 ± 0.4	0.6 ± 0.4
	U(31,28) = 431	*p* = 0.9700
	n.s.	
2nd spike, 1st component AHP (mV)	–9.5 ± 0.8	–9.7 ± 0.5
	U(31,28) = 406	*p* = 0.6786
	n.s.	
2nd spike, 1st AHP component latency (ms)	8.9 ± 0.4	9.4 ± 0.6
	U(31,28) = 368	*p* = 0.3226
	n.s.	
2nd spike, 2nd component AHP (mV)	–15.9 ± 0.6	–16.1 ± 0.4
	U(31,28) = 422	*p* = 0.8625
	n.s.	
2nd spike, 2nd AHP component latency (ms)	47.5 ± 2.3	46.2 ± 2.1
	U(31,28) = 400	*p* = 0.6136
	n.s.	
2nd spike, ADP (mV)	0.0 ± 0.0	0.3 ± 0.3
	U(31,28) = 418.5	*p* = 0.8154
	n.s.	
2nd spike, ADP latency (ms)	0.0 ± 0.0	2.9 ± 0.4
	U(31,28) = 418.5	*p* = 0.8154
	n.s.	

*n, number of cells; n.s., not statistically significant.*

*Mann–Whitney *U* tests and corresponding exact *p*-values.*

GluT1 and GluT3 mRNAs were respectively detected in 29% (*n* = 9 of 31 cells) and 48% (*n* = 15 of 31 cells) in non-Tg neurons and in 18% (*n* = 5 of 28 cells, *p* = 0.3703) and 21% (*n* = 6 of 28 cells, *p* = 0.0555) in 3xTg-AD neurons (**Figures 6B,C**). GluT1 and GluT3 transcripts were rarely co-detected in both non-Tg (23%, *n* = 7 of 31 cells) and 3xTg-AD neurons (4%, *n* = 1 of 28 cells, *p* = 0.0547). Similar proportions of neurons were found to express only GluT1 or only GluT3 in non-Tg and 3xTg-AD neurons (7%, *n* = 2 of 31 cells vs. 14%, *n* = 4 of 28 cells, *p* = 0.4092 for GluT1 and 26%, *n* = 8 of 31 cells vs. 18%, *n* = 5 of 28 cells, *p* = 0.5398 for GluT3, respectively). Finally, we detected at least one of these two GluTs in a similar proportion of non-Tg (55%, *n* = 17 of 31 cells) and 3xTg-AD neurons (36%, *n* = 10 of 28 cells, *p* = 0.1925). These observations are consistent with the similar glucose uptake capacity that we observed in non-Tg and 3xTg-AD layer II and III pyramidal cells (**Figure 2**).

Similarly, the frequency of detection of HK1 was not different between non-Tg (42%, *n* = 13 of 31 cells) and 3xTg neurons (54%, *n* = 15 of 28 cells, *p* = 0.4390). Pfkfb3 was only detected in a minority of pyramidal cells of non-Tg (13%, *n* = 4 of 31 cells) and 3xTg-AD (4%, *n* = 1 of 28 cells, *p* = 0.3563, **Figures 6B,C**). The most frequently detected phosphofructokinase-1 isoforms in both non-Tg and 3xTg-AD neurons were PFK1m (84%, *n* = 26 of 31 cells and 64%, *n* = 18 of 28 cells, *p* = 0.1339, **Figures 6B,C**) and PFK1p (58%, *n* = 18 of 31 cells, and 50%, *n* = 14 of 28 cells, *p* = 0.3598, **Figures 6B,C**). PFK1l was observed in 36% (*n* = 11 of 31 cells) and 29% (*n* = 8 of 28 cells, *p* = 0.3874, **Figures 6B,C**) in non-Tg and 3xTg-AD neurons, respectively.

G6PDx was detected in 16% (*n* = 5 of 31 cells) and 18% (*n* = 5 of 28 cells) of the non-Tg and 3xTg-AD pyramidal cells, respectively (*p* = 1.000, **Figures 6B,C**). The glycogen synthase Gys1 was never detected in non-Tg neurons and rarely in 3xTg-AD neurons (7%, *n* = 2 out of 28, *p* = 0.2209, **Figures 6B,C**). Finally, the glycogen phosphorylase, PygB was observed in similar proportions in non-Tg (29%, *n* = 9 out of 31) and 3xTg-AD pyramidal cells (29%, *n* = 8 out of 28, *p* = 1.000, **Figures 6B,C**).

## Discussion

**FIGURE 7:**
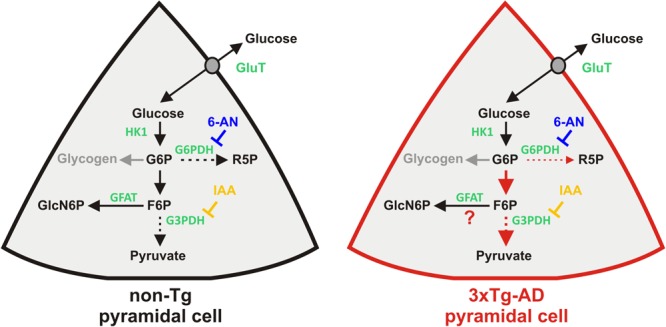
Resting glucose metabolism of juvenile pyramidal cells and its alterations in 3xTg-AD mice. Glucose uptake is facilitated via glucose transporters (GluT). Once in the cytoplasm glucose phosphorylation by Hexokinase1 (HK1) yields to glucose 6-phosphate (G6P), the common substrate of different metabolic pathways. Glucose-6-phosphate dehydrogenase (G6PDH) is the rate limiting enzyme of the pentose phosphate pathway leading to ribose-5-phosphate (R5P). Pentose phosphate pathway is blocked by the G6PDH inhibitor, 6-aminonicotinamide (6-AN). G6P can also be isomerized into fructose-6-phosphate (F6P), a common intermediate of the glycolysis and the hexosamine biosynthetic pathway. Glycolysis is blocked by Iodoacetic acid (IAA) inhibiting glyceraldehyde-3-phosphate dehydrogenase (G3PDH). Glutamine:fructose-6-phosphate amidotransferase (GFAT), the rate limiting enzyme of the hexosamine biosynthetic pathway, converts fructose-6-phosphate and glutamine into glucosamine-6-phosphate (GlcN6P) and glutamate (glutamine and glutamate are omitted for the sake of simplicity). G6P is also the precursor of glycogen whose synthesis is normally absent in neurons (*gray arrow*). *Dashed arrows* indicate multisteps reactions. Functional changes in 3xTg-AD neurons are depicted by *red arrows*. *Thicker* and *thinner* arrows denote increased and decreased pathways, respectively.

Here we studied glucose metabolism of juvenile supragranular pyramidal cells and its early alteration in a mouse model of AD. Using intracellular glucose imaging and scRT-mPCR, we found that glucose uptake was effective in juvenile pyramidal cells and was not altered in 3xTg-AD mice. We also observed that both glycolysis and pentose phosphate pathway were active under resting condition. Interestingly, in 3xTg-AD neurons the pentose phosphate pathway was reduced in favor of glycolysis (**Figure 7**). IAA application also disclosed that another glucose metabolic pathway might be active in 3xTg-AD neurons. The scRT-mPCR analysis allowed to establish the three expression profile of glucose metabolism key genes in pyramidal neurons, which appeared qualitatively unaltered in 3xTg-AD mice. This indicates that the metabolic alterations observed in 3x-Tg-AD pyramidal neurons are possibly due to post-transcriptional modifications.

### Intracellular Glucose Imaging in Pyramidal Cells

We visualized the dynamics of intracellular glucose level using a genetically encoded biosensor exhibiting changes in FRET upon glucose binding but insensitive to phosphorylated glucose or glycolytic intermediates (Fehr et al., 2003; Deuschle et al., 2005; John et al., 2008; Takanaga et al., 2008; Bittner et al., 2011; Hou et al., 2011; Barros et al., 2013). The glucose sensor FLII12Pglu-700 μδ6 was transduced using a Sindbis vector which exhibits a strong tropism toward pyramidal cells (Chen et al., 2000; Lendvai et al., 2000; Furuta et al., 2001; Drobac et al., 2010). Consistently, a large majority of transduced cells were immunopositive for Satb2 (**Figure 1**), a transcription factor of pyramidal cells (Britanova et al., 2008; Tasic et al., 2016). Given that not all upper-layer pyramidal cells express Satb2 (Britanova et al., 2008; Tasic et al., 2016), the proportion of excitatory neurons expressing the glucose sensor is likely to be higher. Thus, our glucose imaging experiments performed in layer II and III largely reflects the metabolic activity of upper layer pyramidal cells.

### Glucose Uptake

In addition to GluT3, a well established neuronal glucose transporter (Nagamatsu et al., 1992; McCall et al., 1994; Fields et al., 1999), we also detected GluT1 in a minority of pyramidal cells. Although, GluT1 is more classically described in glial and endothelial cells (Kacem et al., 1998; McEwen and Reagan, 2004; Simpson et al., 2007; Winkler et al., 2015), recent transcriptomic studies have revealed GluT1 expression in neurons (Cahoy et al., 2008), including layer II and III cortical pyramidal cells (Tasic et al., 2016). These observations indicate that juvenile supragranular pyramidal cells express GluT3 and/or GluT1 transcripts.

Intracellular glucose imaging revealed that juvenile pyramidal cells have the capacity to take up glucose (**Figure 2**). This is consistent with the relatively low rate of glucose uptake reported in resting cortical neurons (Chuquet et al., 2010; Diaz-Garcia et al., 2017). Furthermore, we observed a low occurrence of GluT1 and GluT3 mRNAs (**Figure 6**), in agreement with the low expression level of these transporters in rodents during the first postnatal weeks (Khan et al., 1999; Vannucci and Simpson, 2003). Since scRT-mPCR sensitivity is limited by the abundance of transcripts and by the amount of cytoplasm collected (Cauli and Lambolez, 2010; Devienne et al., 2018), this technique barely detects transcripts expressed at a very low single cell level. This could explain the relatively low level of GluT1 and GluT3 detection in pyramidal cells despite a glucose uptake capacity that we observed in virtually all analyzed neurons. In addition, since the proportion of neurons expressing at least one GluT is higher than that of those expressing a given GluT, a lack of GluT1 expression is likely to be functionally compensated by expression of GluT3 and vice versa. Along this line, functional compensation of glucose transport could be also achieved by other glucose transporters such as GluT6 whose transcripts have been consistently reported in cortical neurons (Cahoy et al., 2008; Tasic et al., 2016). Altogether, our observations indicate that at this juvenile stage, when energy metabolism is still heavily dependent on ketone bodies (Nehlig, 1999), pyramidal cells can already use glucose.

Neither GluT1 and 3 expression nor glucose uptake capacity was altered in juvenile 3xTg-AD pyramidal cells. Indeed, quantitative impairments in GluTs expression and glucose utilization have only been reported after 6 months of age in 3xTg-AD mice (Nicholson et al., 2010; Chen et al., 2012; Ding et al., 2013).

### Glucose Fate in Juvenile Pyramidal Cells

HK1, the predominant brain hexokinase isoform (Wilkin and Wilson, 1977; Fields et al., 1999; Khan et al., 1999) was detected in a substantial proportion of pyramidal neurons confirming its expression at a relatively low level in this cell type (Tasic et al., 2016). This suggests that juvenile pyramidal cells have already the ability to metabolize glucose into glucose-6 phosphate as indicated by our glucose imaging observations (**Figures 3, 7**).

As previously observed (Zeitschel et al., 1996; Bigl et al., 2003; Cahoy et al., 2008; Tasic et al., 2016), the key enzymes of glycolysis PFK1m and PFK1p, but also FPK1l to a lesser extent, were frequently detected in juvenile pyramidal cells. In contrast, pfkfb3 was rarely detected (Tasic et al., 2016). These molecular data indicate that pyramidal cells are well equipped for glycolysis. To evaluate functionally glycolysis fluxes in slices, we applied 200 μM IAA for 30 min. Such a treatment achieves an efficient glycolysis inhibition reducing NADH level within 5 min (Gordon et al., 2008) in agreement with IAA-induced G3PDH inhibition, all the while minimizing unspecific effects (Dringen et al., 1993). Blockade of the glycolytic flux generates a progressive accumulation of glucose-6-phosphate (Bittner C.X. et al., 2010) which in turn strongly inhibits HK1 (Liu et al., 1999), leading to an intracellular glucose increase (John et al., 2008). During IAA exposure, we consistently observed a delayed accumulation of intracellular glucose. Confirming a recent report (Diaz-Garcia et al., 2017), our results collectively reveal an active glycolysis in juvenile pyramidal cells (**Figures 4A, 7**).

G6PDx is a major rate limiting enzyme of the pentose phosphate pathway. In agreement with previous observations (Ninfali et al., 1998; Cahoy et al., 2008; Tasic et al., 2016), we detected its transcripts suggesting that juvenile pyramidal cells can also use glucose for the pentose phosphate pathway. To determine functionally the activity of this metabolic pathway we used 6-AN, an inhibitor of two key enzymes, G6PDH and 6-phosphogluconate dehydrogenase (Lange and Proft, 1970; Kohler et al., 1970) which has a mild impact on glycolysis in the short term (Hothersall et al., 1981; Haghighat and McCandless, 1997). Blockade of the pentose phosphate pathway with 6-AN induced an increase of intracellular glucose in non-Tg juvenile pyramidal cells (**Figures 4B, 7**). Taken together, our data indicate that pentose phosphate pathway is active in these neurons.

Under physiological conditions, glycogen is absent from neurons (Vilchez et al., 2007). Consistent with previous reports (Zeisel et al., 2015; Tasic et al., 2016), the glycogen synthase (Gys1), and the glycogen phosphylase (PygB), were rarely detected in non-Tg pyramidal cells.

Overall our molecular and imaging data indicate that the glycolysis and the pentose phosphate pathway are the two main active glucose pathways in juvenile pyramidal cells (**Figure 7**).

### Alteration of Glucose Metabolic Pathways in 3xTg-AD Pyramidal Cells

We observed a higher glycolytic activity and a less active pentose phosphate pathway in 3xTg-AD juvenile pyramidal neurons (**Figure 7**). Interestingly a NMR study has reported an increased neuronal glycolysis in 7-months old 3xTg-AD mice (Sancheti et al., 2014b) indicating that the higher glycolytic activity develops early and persists before aging (Sancheti et al., 2014a). Consistently, a recent metabolomic study revealed a higher lactate plasma level in 8-months old 3xTg-AD mice (Sanguinetti et al., 2018) that could result from a higher neuronal glycolytic flux (Diaz-Garcia et al., 2017).

Our molecular observations did not reveal any significant change in the expression profile of key molecular elements of glucose metabolism in 3xTg-AD juvenile pyramidal cells. However given the detection limits of the scRT-mPCR discussed above and the relatively low occurrence G6PDx transcripts, it is possible that the lower pentose phosphate pathway observed in 3xTg-AD mice derives from a reduction of G6PDx expression. To our knowledge, changes in the expression profile of key molecular elements of glucose metabolism in 3xTg-AD mice have only been reported after 6 months of age (Chen et al., 2012; Ding et al., 2013). This suggests that the early alterations of metabolic activity are likely mediated by post-transcriptional and/or post-translational modifications. Interestingly, microRNA-34a overexpression reduces G6PDH protein level and was found to be increased in the temporal cortex of both 3xTg-AD mice and AD patients (Sarkar et al., 2016). Similarly, the activity of several key elements controlling glucose pathways are regulated at the post-translational level (Khan et al., 1999; Almeida et al., 2004; Vilchez et al., 2007; Bolanos et al., 2008; Herrero-Mendez et al., 2009).

Given the low level of detection of pfkfb3 (**Figure 6**; Tasic et al., 2016), it is unlikely that 3xTg-AD pyramidal cells upregulate glycolysis via fructose 2,6-bisphosphate production (Almeida et al., 2004; Herrero-Mendez et al., 2009). Exposure to Aβ peptides has been shown to increase the glycolytic activity of astrocytes (Allaman et al., 2010). It would be interesting to determine whether Aβ peptides, even at very low level, could be responsible for the early alterations we observed in 3xTg-AD pyramidal cells. Further investigations will be needed to elucidate the underlying mechanisms.

Upon IAA application, we observed a transient increase in glucose utilization in 3xTg-AD pyramidal cells. A rerouting of glucose utilization toward the pentose phosphate pathway and/or glycogenesis is unlikely given that the increased glucose consumption induced by IAA persisted in presence of 6-AN (**Figure 4C**) and that pyramidal neurons rarely express glycogen synthase (**Figure 6**; Zeisel et al., 2015; Tasic et al., 2016). An alternative possibility would be an increased glucose utilization via the hexosamine biosynthetic pathway which competes with glycolysis for fructose-6-phosphate (**Figure 7**). The entry in this metabolic pathway is controlled by the rate limiting enzyme glutamine:fructose-6-phosphate amidotransferase (GFAT), which converts fructose-6-phosphate and glutamine to glucosamine-6-phosphate and glutamate (Buse, 2006). Interestingly, 3xTg-AD mice exhibit a temporary increased brain level of glutamine (Sancheti et al., 2014a,b) that could account for its higher plasma level (Sanguinetti et al., 2018). Thus, upon glycolysis inhibition, the hexosamine biosynthetic pathway could be favored by an enhanced availability of the two GFAT substrates in these mice. Indeed, IAA will induce a retrograde increase of the glycolysis intermediaries before HK inhibition by glucose-6-phosphate (Liu et al., 1999) and subsequent glucose accumulation (John et al., 2008). Thus fructose-6-phosphate would be then transiently available to feed the hexosamine biosynthetic pathway. Glutamine synthesis largely relies on astrocytes metabolism. In slices, IAA application would also affect astrocytes glycolysis and would lead to a shortage in glutamine production. Therefore, an enhanced flux of the hexosamine biosynthetic pathway in 3xTg-AD pyramidal cells is expected to be transient. This hypothesis is compatible with the fast inhibition of G3PDH by IAA in astrocytes (Gordon et al., 2008) and the kinetics of the transient decrease in intracellular glucose observed (**Figure 4**).

Interestingly, the hexosamine biosynthetic pathway is essential for *O*-*N*-acetylglucosamine glycosylation, a process that protects tau protein against hyperphosphorylation (Liu et al., 2009; Gatta et al., 2016). An increased activity of this pathway might represent an early adaptive mechanism to prevent and/or delay neurofibrillary tangles formation in pyramidal cells. The later impairment of glucose uptake (Nicholson et al., 2010; Ding et al., 2013) and decreased levels of glutamine (Sancheti et al., 2014a) observed in 3xTg-AD mice is likely to reduce the activity of the hexosamine pathway leading to tau hyperphosphorylation and tangles formation (Oddo et al., 2003; Carroll et al., 2010; Gatta et al., 2016).

## Conclusion

Despite a low cerebral glucose metabolism in suckling rodents (Nehlig, 1999), its qualitative alterations in 3xTg-AD pyramidal cells at a very early stage may have deleterious consequences for their activity and survival in the long-term. Indeed, neurons are poorly equipped to detoxify methylglyoxal, a harmful glycolysis byproduct (Belanger et al., 2011). An increased glycolytic activity is therefore expected to alter pyramidal cells survival (Herrero-Mendez et al., 2009). Similarly, a decreased pentose phosphate pathway would reduce the capacity of neurons to produce glutathione, impairing their ability to detoxify reactive oxygen species (Vaughn and Deshmukh, 2008). A higher glycolysis activity combined with a reduced pentose phosphate pathway would dramatically increase pyramidal cell vulnerability. This could contribute to their degeneration which occurs before amyloid deposition (Bittner T. et al., 2010). Further investigations will be required to determine how these metabolic alterations evolve during maturation and to identify their underlying mechanisms, which could provide potential targets for drug-modifying therapies.

## Author Contributions

BC, JP, and GB conceived and designed the experiments. JP, XT, JLD, ÉF, and BC performed the experiments. JP and BC analyzed the data. JP, BC, and GB interpreted the results. RH, RL, and EG contributed reagents and materials. JP, BC, and GB wrote the manuscript.

## Conflict of Interest Statement

The authors declare that the research was conducted in the absence of any commercial or financial relationships that could be construed as a potential conflict of interest.

## Footnotes

1 http://fiji.sc/Fiji
